# Why and how do dual users quit vaping? Survey findings from adults who use electronic and combustible cigarettes

**DOI:** 10.18332/tid/132547

**Published:** 2021-02-16

**Authors:** Elias M. Klemperer, Andrea C. Villanti

**Affiliations:** 1 Vermont Center on Behavior and Health, Department of Psychiatry, University of Vermont, Burlington, United States; 2 Department of Psychological Science, University of Vermont, Burlington, United States

**Keywords:** electronic cigarettes, cigarette smoking, quit attempt, cessation, motivation to quit

## Abstract

**INTRODUCTION:**

Most adults who use electronic cigarettes (ECs) also smoke combustible cigarettes (CCs). Quitting ECs appears common among dual users but little is known regarding adult dual users’ motivations and methods to quit ECs or how this relates to quitting CCs.

**METHODS:**

We used Amazon Mechanical Turk, a web-based crowd-sourcing service, to survey 366 US adults with a history of regular EC and CC use. This analysis examined motivations and methods to quit both products among a subset of 204 (55.7%) respondents with dual use and a history of one or more attempts to quit ECs.

**RESULTS:**

Most respondents (95%) were using ECs at the time of this survey and had a lifetime median of five EC quit attempts. The most common motivations to quit ECs were health (74%), money/cost (45%), and to reduce risk of COVID-19 (25%). The most common EC quit methods were cutting down (68%), getting advice from a doctor (28%), quitting 'cold turkey' (24%), nicotine replacement therapy (24%), and switching to ECs with less nicotine (24%). Most motivations and methods to quit ECs and CCs were moderately to highly correlated, suggesting similarity in dual users’ approach to quitting the two products.

**CONCLUSIONS:**

Dual users had a range of motivations and methods to quit ECs, most of which were similar to their motivations and methods to quit CCs. These findings support the need to develop treatment for adults motivated to quit ECs and demonstrate that dual users may currently engage in similar strategies to quit both products.

## INTRODUCTION

Electronic cigarette (EC) use has increased dramatically in the past decade^1^. Though initiating ECs to quit combustible cigarettes (CCs) is common^1^, most adults who use ECs continue smoking CCs^1,2^. Among those who continue to smoke CCs (i.e. dual users), motivation to stop using both products appears common^3-5^. Most US adults (62%) who use ECs plan to quit ECs at some point in the future^6^ and 45% of US adolescents who use ECs report seriously thinking about quitting ECs^7^. However, little is known regarding the specific reasons (i.e. motivations) and strategies used (i.e. methods) to quit ECs among adults who use both ECs and CCs.

Discontinuing EC use is common among adult CC smokers who try using ECs^3-5^. In a large national study of US adults, over half (56%) of current CC smokers who started using ECs later discontinued ECs^4^ and findings were similar in a European cohort study with a two year follow-up^5^. Much of the prior research on stopping ECs includes adults who tried and subsequently discontinued ECs in the absence of self-defined regular EC use or dependence^4,8^. Among adults with a history of regular EC use, the most common reasons for discontinuation were that ECs were not rewarding^9,10^ or useful^9,11^ enough to warrant continued use.

In contrast, people who quit nicotine products often do so because of competing contingencies (e.g. health concerns, stigma, or monetary cost) despite continued cravings or urges to smoke. For example, among adults who smoke CCs, quitting often requires motivation^12^, multiple failed attempts^13^, and treatment^14^ to overcome symptoms of CC dependence and withdrawal. Use of ECs can also produce dependence^1^ and withdrawal upon cessation^15^. Thus, among those who become dependent on ECs, quitting ECs could require some motivations or strategies similar to quitting CCs^16^. Though research on reasons for quitting ECs among adults is limited, one recent qualitative study identified health, financial cost, and freedom from addiction as common reasons for wanting to quit ECs among youth and young adults seeking EC cessation treatment^17^ but did not assess reasons for quitting among dual users *per se*. Further, little is known regarding the influence of the ongoing COVID-19 pandemic on motivations to quit ECs.

With regard to methods for quitting ECs, one recent analysis identified that approximately half of US adults with an attempt to quit ECs in the past year attempted to quit gradually (i.e. reduced to quit)^6^. Among adult EC users who attempted to quit all at once, common strategies included support from family or friends (25%) and counseling or self-help materials (11%). Though 55% of respondents were also current CC smokers, this study did not distinguish between EC cessation strategies used by dual users versus exclusive EC users^6^. Methods used to quit ECs could be limited by the lack of empirically supported EC cessation treatment options. A text-message based EC cessation service has engaged a large number of youth and young adult EC users^3^, for which a randomized controlled effectiveness trial is ongoing^18^. A non-randomized exploratory study offered a sample of dual users varenicline and found that self-selected use of the medication was a strong predictor of prolonged EC abstinence at a 6-month follow-up (OR=7.8)^19^. Examples of quitting ECs include a case study which documented the use nicotine replacement therapy (NRT) to quit ECs^20^ and another case study that described the use of motivational interviewing in combination with nicotine tapering to quit ECs^21^.

There is a dearth of empirically-supported treatments for EC cessation among adults and little is known about how dual users attempt to quit ECs on their own (i.e. in the absence of researcher or healthcare provider intervention). Further, it is unclear whether adult dual users have similar or different motivations for attempting to quit ECs versus CCs. We surveyed adults with a history of dual use (i.e. regular use of both ECs and CCs) who had attempted or succeeded at quitting ECs to improve understanding of motivations and methods to quit ECs and how this relates to quitting CCs.

## METHODS

We recruited participants using Amazon Mechanical Turk, a web-based crowd-sourcing service. Eligibility criteria were: 1) aged ≥21 years, 2) live in the US, 3) a lifetime history of use of ECs containing nicotine on ≥15 days in a given month, 4) CC smoking on ≥15 days in a given month, and 5) one or more attempts to reduce or quit ECs in their lifetime. We elected to exclude people with EC and CC use on <15 days in a given month in order to ensure that included participants were regular users of nicotine products (i.e. ≥50% of days). Of the 593 who screened, 366 were eligible and completed the survey in April 2020. The analytic sample for this study comprised a subset of 204 (55.7%) respondents who reported a history of simultaneous regular use of both ECs and CCs and a history of one or more attempts to quit ECs (See Supplementary file Table S1 for a description of participants excluded from this analysis). The survey was originally designed to inform a tobacco treatment intervention for dual users that will be tested in an upcoming randomized controlled trial. This study was approved by the Institutional Review Board at the University of Vermont and all respondents consented to complete this survey.

### Measures

#### Demographics and tobacco use

Respondents answered questions regarding demographics and past 30 days EC devices and flavors used, frequency of EC and CC use, and time to first EC or CC use after waking. Time to first CC is a validated and commonly used indicator of cigarette dependence^22^ and time to first EC appears to be a valid measure of EC dependence^23^. We assessed attempts to quit by asking: ‘How many times in your life have you intentionally [“quit vaping nicotine or quit using e-cigarettes” OR “quit smoking cigarettes”] for a day (24 hours) or more?’. We considered respondents to be currently abstinent from ECs or CCs if they responded ‘0’ to ‘During the past 30 days, on how many days did you [“use an e-cigarette/ vape containing nicotine” OR “smoke one or more cigarettes”]?’.

#### Motivations to quit

We assessed respondents’ motivations to quit ECs and CCs separately by asking: ‘What were your reasons for quitting or attempting to quit [“e-cigarettes/vapes containing nicotine” OR “smoking”]?’. We instructed respondents to ‘select all that apply’ from a list of common motivations to quit each product adapted from ongoing EC research^24^: a) health; b) money/ cost; c) didn’t like the taste or smell, d) difficulty obtaining [‘device, e-liquid, pods, cartridges’ OR ‘cigarettes’]; e) negative experience while using; f) I was embarrassed by my [‘e-cigarette/vape use’ OR ‘smoking’]; g) to reduce the risk of harm from coronavirus (COVID-19); h) I started or increased e-cigarette use or vaping [offered as a motivation to quit CCs only], i) to perform better in school, work, sports, etc.; j) for another person, like a family member or friend or partner; k) I started or increased other tobacco use; l) I didn’t like [‘e-cigarettes or vaping’ OR ‘smoking cigarettes’] anymore; and m) freedom from addiction.

#### Methods to quit

We assessed methods for quitting each product separately by asking: ‘What methods have you used to quit [“e-cigarettes/vapes containing nicotine” OR “smoking cigarettes”]’?. We instructed respondents to ‘select all that apply’ from a list of common methods adapted from prior research^25^: a) cut down on my [‘e-cigarette/vape use’ OR ‘smoking’] before quitting; b) switched to pods, liquids or e-juice with less nicotine before quitting (asked for quitting ECs only); c) switched to e-cigarettes/vapes containing nicotine (asked for quitting CCs only); d) got advice from my doctor on quitting; e) used nicotine patch, gum or lozenge; f) used bupropion or Zyban; g) used varenicline or Chantix; h) called a telephone quitline; i) used an app on my phone, smart-watch, tablet, or computer; j) read written materials on quitting; i) went to individual or group counseling for help with quitting; k) ‘cold turkey’ (quit abruptly without help); l) switched to a different tobacco product; and m) other. In addition, we instructed respondents to select the single method that was ‘most effective to help you quit’.

#### Additional quitting measures

We assessed change in CC smoking during respondents’ last EC quit attempt by asking: ‘Which of the following is true about the last time you quit e-cigarettes/vapes containing nicotine?’: a) I didn’t smoke cigarettes before or after I quit e-cigarettes/ vapes; b) I smoked cigarettes about the same before and after I quit e-cigarettes/vapes; c) My cigarette smoking decreased when I quit cigarettes/vapes; d) My cigarette smoking increased when I quit e-cigarettes/vapes; and e) I don't know’. We used the same language to ask about change in EC use the last time respondents quit CCs. We assessed respondents’ perception of their EC use when quitting CCs by asking: ‘How did using e-cigarettes/vapes affect quitting cigarette smoking? (0=made it much easier to quit smoking; 10=made it much harder to quit smoking)’.

### Statistical analysis

We used descriptive statistics to summarize demographic information, tobacco use characteristics, and methods and motivators for quitting each product. In addition, we calculated point-biserial correlations between motivations and methods to quit ECs and CCs. There were no missing data for methods or motivations to quit ECs. With regard to CCs, nine respondents were missing data for methods used to quit and none were missing data for motivations to quit CCs. We conducted all analyses using SPSS (IBM Corp., Armonk, NY, USA). Missing data were handled with listwise deletion per SPSS default procedures.

## RESULTS

Respondents were mostly White, male, who were in their mid thirties (Table 1). All had attempted to quit both ECs and CCs and few (<5%) achieved abstinence from either product. At the time of this survey, respondents were primarily non-daily users of ECs and CCs and most used tobacco flavored rechargeable pod-based ECs in the past 30 days (Table 1). On the days they used ECs or CCs, over a quarter of respondents vaped or smoked within 30 minutes of waking.

**Table 1:** Participant characteristics (N=204)

*Characteristics*	*% (95% CI)*
**Female**	30.9 (24.9–37.6)
**Age** (years), Mean ± SD	35.7 ± 10.5
**Marital status**	
Married or living as if married	71.1 (64.4–76.9)
Widowed, divorced, separated	5.4 (3.0–9.5)
Never married	23.5 (18.2–29.9)
**Education level**	
<High school	4.4 (2.3–8.3)
High school graduate or GED	9.8 (6.0–14.2)
Some college or Associate’s degree	17.6 (13.0–23.5)
Bachelor’s or advanced degree	68.1 (61.4–74.2)
**Hispanic ethnicity**	23.0 (17.7–29.4)
**Race^a^**	
American Indian/Alaska Native	3.9 (2.0–7.7)
Asian	2.0 (0.7–5.1)
Black/African American	11.3 (7.6–16.4)
Native Hawaiian/Pacific Islander	1.0 (0.2–3.9)
White	84.8 (79.2–89.1)
**Employment**	
Full-time ≥35 h/week	91.7 (87.0–94.8)
Part-time <35 h/week	5.9 (3.4–10.2)
Currently not working for pay	2.5 (0.7–5.2)
**Median age of first use** (years)	
ECs	20
CCs	18
**Median number of lifetime quit attempts**	
ECs	5
CCs	5
**Current 30-day abstinence**	
Abstained from ECs only	2.9 (1.3–6.4)
Abstained from CCs only	2.9 (1.3–6.4)
Abstained from both ECs and CCs	2.0 (0.7–5.1)
**Current dual users** (n=194)	
**Days of use in past 30 days,** Mean ± SD	
ECs	17.7 ± 8.9
CCs	18.9 ± 9.4
**EC device used in past 30 days^a^**	
Rechargeable or pod-based device	73.7 (67.0–79.5)
Disposable device	17.0 (12.3–23.0)
Modular device with refillable tank	6.7 (3.9–11.2)
Other or not sure	2.6 (1.1–6.1)
**EC flavor used in past 30 days^a^**	
Tobacco	66.5 (59.5–72.8)
Menthol	46.4 (39.4–53.5)
Mint	42.3 (35.5–49.4)
Clove or spice	8.8 (5.5–13.7)
Fruit	24.2 (18.7–30.8)
Chocolate	25.8 (20.1–32.4)
Alcoholic drink	15.5 (11.0–21.3)
Non-alcoholic drink	6.7 (3.9–11.2)
Candy or sweets	14.9 (10.6–20.7)
Other	4.6 (2.4–8.7)
**Used within 30 minutes of waking**	
ECs	31.4 (25.2–38.4)
CCs	27.8 (21.9–34.6)

a Respondents could select one or more options.

CC: combustible cigarette. EC: electronic cigarette. GED: General Education Development. SD: standard deviation. All participants from the USA and completed the Amazon Mechanical Turk survey for this study in April 2020.

### Motivations to quit

**Figure 1:**
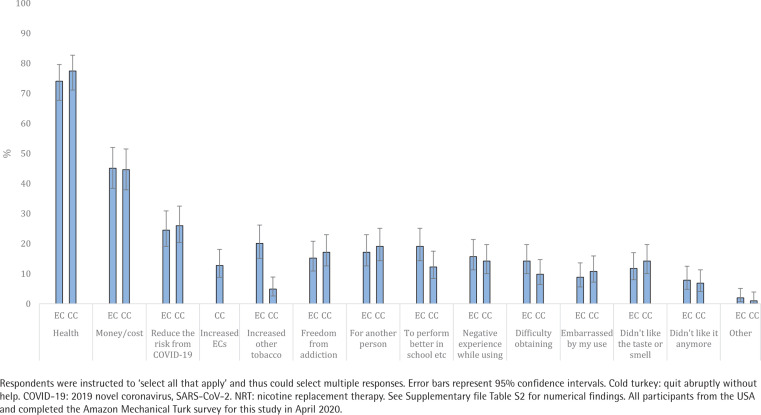
Motivations for quitting e-cigarettes (EC) and combustible cigarettes (CC) among dual users (N=204)

Respondents endorsed a median of two reasons (of the 13 response options) for quitting ECs. The most common were: 1) health, 2) money/cost, 3) to reduce the risk of harm from COVID-19, 4) increased use of other tobacco, and 5) to perform better at work or school (Figure 1 and Supplementary file Table S2). Similarly, respondents endorsed a median of two reasons (of the 14 response options) for quitting CCs. The most common were: 1) health, 2) money/cost, 3) to reduce the risk of harm from COVID-19, 4) freedom from addiction, and 5) for another person. There were moderate to large correlations between quitting ECs and CCs for 12 of the 13 reasons that applied to both products (r_pb_ range: 0.35–0.65; Supplementary file Table S3). The most highly correlated motivations for quitting were to reduce risk of harm from COVID-19 (r_pb_=0.65, p<0.001), for another person (r_pb_=0.61, p<0.001), and money/cost (r_pb_=0.57, p<0.001). See Table 2 for the motivations to quit endorsed by the 10 respondents who achieved 30-day abstinence from ECs and the 10 respondents who achieved 30-day abstinence from CCs.

**Table 2:** Motivations and methods to quit electronic cigarettes (ECs) and combustible cigarettes (CCs) among the respondents abstinent from ECs or CCs for the past 30 days with a history of dual use

	*ECs (n=10)*	*CCs (n=10)*
**Motivation to quit**		
Health	6	9
Money/cost	6	6
Reduce the risk from COVID-19	0	1
Increased ECs	0	1
Increased other tobacco	3	0
Freedom from addiction	0	4
For another person	0	1
To perform better in school etc.	0	0
Negative experience while using	1	1
Difficulty obtaining	0	0
Embarrassed by my use	3	2
Didn’t like the taste or smell	2	0
Didn’t like it anymore	3	1
Other		
**Method used to quit**		
Cut down before quitting	5	7
Cold turkey*	7	2
Got advice from doctor	1	2
Switched to ECs	0	2
Switched to low nicotine pods	0	0
Used NRT	1	3
Read written material	0	1
Used an app	1	1
Went to counseling	0	2
Switched to different tobacco	3	0
Used bupropion	0	0
Used varenicline	0	0
Called a quitline	0	0
Other	0	0

Respondents were instructed to ‘select all that apply’ and thus could select multiple responses.

* Cold turkey: quit abruptly without help.

COVID-19: 2019 novel coronavirus, SARS-CoV-2. NRT: nicotine replacement therapy. All participants from the USA and completed the Amazon Mechanical Turk survey for this study in April 2020.

### Methods used to quit

**Figure 2:**
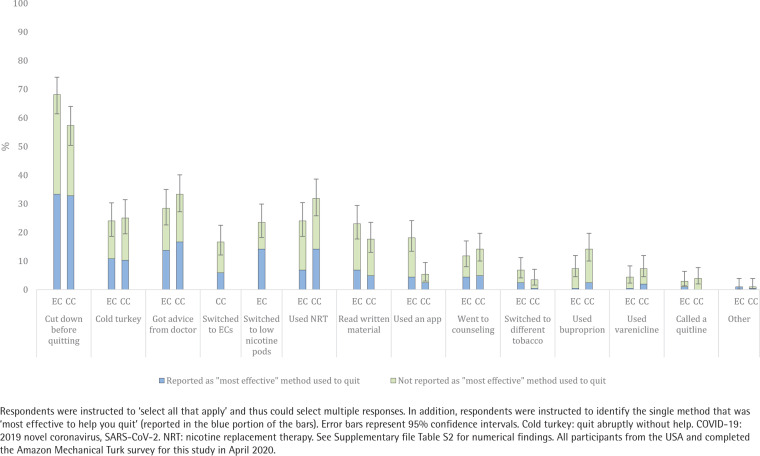
Methods used to quit e-cigarettes (EC) and combustible cigarettes (CC) among dual users (N=204)

Respondents endorsed a median of two strategies (of the 13 response options) used to quit ECs. The most common were: 1) cutting down to quit (i.e. reduction), 2) quitting ‘cold turkey’ (i.e. quit abruptly without aid), 3) getting advice from a doctor, 4) NRT, and 5) switching to ECs with less nicotine (Figure 2 and Supplementary file Table S2). Respondents reported that the most effective methods to quit ECs were cutting down first (35.3%), quitting ‘cold turkey’ (15.8%), and switching to ECs with less nicotine (14.2%; Figure 2 and Supplementary file Table S2). Similarly, respondents endorsed a median of two strategies (of the 13 response options) used to quit CCs. The most common were: 1) cutting down to quit, 2) getting advice from a doctor, 3) quitting ‘cold turkey’, 4) NRT, and 5) switching to ECs or reading written material. Respondents reported the most effective methods to quit CCs were cutting down first (36.8%), getting advice from a doctor (17.7%), and using NRT (16.2%; Figure 2 and Supplementary file Table S2). There were moderate to large correlations between 10 of the 12 strategies used to quit that applied to both ECs and CCs (r_pb_ range: 0.35–0.60; Supplementary file Table S4). The most highly correlated methods used to quit were reading written material (r_pb_=0.60, p<0.001), using bupropion (r_pb_=0.58, p<0.001), and cutting down to quit (r_pb_=0.58, p<0.001). See Table 2 for the methods to quit endorsed by the 10 respondents who achieved 30-day abstinence from ECs and the 10 respondents who achieved 30-day abstinence from CCs.

### Additional quitting outcomes

During respondents’ most recent attempt to quit ECs, 34.8% (95% CI: 28.5–41.6) decreased their CC smoking, 36.3% (95% CI: 29.9–43.1) did not change their CC smoking, 20.1% (95% CI: 15.1– 26.2) increased their CC smoking, and 8.3% (95% CI: 5.2–13.0) maintained abstinence from CCs. During respondents’ most recent attempt to quit CCs, 36.8% (95% CI: 30.4–43.6) decreased their EC use, 33.8% (95% CI: 27.6–40.6) did not change their EC use, 17.6% (95% CI: 13.0–23.5) increased their EC use, and 5.9% (95% CI: 3.4–10.1) maintained abstinence from ECs. When asked how using ECs affected quitting CCs, respondents reported a mean difficulty of 6.1 (SD=2.6) where: 0=ECs made it much easier to quit CCs; and 10=ECs made it much harder to quit CCs.

## DISCUSSION

Most of the adult dual users in our sample reported multiple motivations to quit ECs and most motivations to quit ECs and CCs were moderately or highly correlated. The similarities between motivations to quit is noteworthy given the differences between products (e.g. combusted vs non-combusted), but not wholly unexpected given that both products deliver nicotine. Health concerns were the most common motivation to quit ECs, which is consistent with prior research on youth and young adults seeking EC cessation treatment^17^ but differs from prior studies that found lack of satisfaction was a primary reason for discontinuing EC use^9,11^. In contrast, we found less than 10% reported motivation to quit because they ‘didn’t like’ ECs anymore. Differences in findings are likely because we assessed motivations to quit among dual users with a history of regular EC use. Much of the prior research assessed discontinuation among participants with past but not current EC use, many of whom no longer found ECs enjoyable. The large correlation between quitting ECs and CCs due to health concerns identified in this study could reflect the growing misperception that ECs are equally or more harmful than CCs^26^. In addition to health concerns, over 40% reported cost was a motivation to quit ECs, which is consistent with prior research on EC discontinuation^9^ and quitting^17^. Finally, though the effects of vaping on risk of harm from COVID-19 are unclear^27^, nearly a quarter of respondents reported that they were motivated to quit ECs to reduce risk of harm from COVID-19. While this motivation to quit overlaps with general health concerns, our findings suggest the coronavirus pandemic may represent a novel influence on nicotine and tobacco use among dual users^28^.

Respondents used a variety of strategies to quit ECs and most tried more than one method of quitting. Cutting down to quit was most commonly endorsed as the single most effective strategy to quit ECs and used by the majority of dual users in this study. This is consistent with national data on adult EC users^6^ and CC smokers^29^ suggesting reduction is among the most common strategies used to quit each product. Thus, reduction-based interventions could be an acceptable form of treatment for people who use both ECs and CCs. Over a quarter of respondents reported getting advice from a doctor to quit, which is consistent with prior research on the importance of medical advice when quitting CCs^30^. This finding highlights the need for research to inform physicians who are tasked with providing cessation advice to adult dual users. Further, nearly a quarter reported switching to low nicotine ECs to quit, which is noteworthy given the U.S. Food and Drug Administration’s authority to regulate the nicotine content of tobacco products, including ECs^31^. Prior research demonstrates that switching to low nicotine CCs decreases CC smoking and dependence^32^ but more research is needed to identify the effects of switching to low nicotine ECs. Nearly a quarter of respondents reported quitting ‘cold turkey’ (i.e. stopping abruptly without help), which may reflect the current lack of available evidence-based treatments for EC cessation. However, a similar number of respondents reported using NRT to quit ECs despite the current lack of evidence to support NRT for EC cessation. There were medium to large correlations between most strategies used to quit ECs and CCs suggesting that dual users with a history of attempts to quit both products may take similar approaches to quitting each product. Future research is needed to assess whether dual users view ECs versus CCs as their ‘primary’ nicotine product and whether this influences strategies used to quit one or both products.

During respondents’ most recent attempt to quit ECs, over one-third decreased their CC smoking and over 20% increased their CC smoking. Similarly, over one-third decreased their EC use and nearly 20% increased EC use during their last attempt to quit CCs. Most (95%) respondents failed to quit ECs, thus the effects of successfully quitting versus continuing ECs on CC smoking remain unclear. The effects of EC cessation on CC smoking could be influenced by how (e.g. quitting ECs with or without treatment) and when (e.g. quitting ECs before, during, or after quitting CCs) dual users quit. Though the effects of quitting ECs are unclear, motivation to quit ECs appears common^6,7^. Thus treatment is needed to maximize potential benefits (e.g. abstinence from all nicotine and tobacco products) and reduce potential harms (e.g. compensatory CC smoking) among dual users who intend to quit ECs.

### Limitations

Findings are from a sample of Mechanical Turk workers with a history of attempts to quit, and thus may not be representative of dual users in the US^33^. The survey assessed past 30 days EC and CC use and thus we were unable to test tobacco use or dependence as predictors of motivations or methods associated with prior quit attempts. The language used to assess motivations to quit in this survey differed from some prior surveys that assessed reasons for discontinuation^9,11^ and thus may not be directly comparable. We assessed health risks as a motivation to quit ECs and CCs independently but did not ask about perceived risks of one product relative to the other. The misperception that ECs are equally or more harmful than CCs could have influenced respondents’ motivations to quit. Given the relatively small sample size, we were unable to test differences between age groups or EC device. We did not collect information regarding why participants initiated EC and CC use or which product participants used first. This survey relied on retrospective recall and thus is subject to recall bias. Findings are cross-sectional and do not identify the longitudinal effects of quitting ECs.

## CONCLUSIONS

This survey identified a range of motivations and methods to quit ECs among adult dual users and highlights the need for longitudinal research on attempts to quit ECs. Most respondents had more than one reason and tried more than one strategy to quit. Health was the most common motivation to quit ECs and cutting down to quit was the most common method. Most reasons and strategies to quit ECs and CCs were moderately to highly correlated, suggesting a similarity in dual users’ views on quitting the two products despite the fact the CCs are combusted and ECs are not. Broadly, these findings support the need to develop treatment for adult dual users motivated to quit ECs and demonstrate dual users could engage in similar strategies to quit both products.
