# Supplementary material for: Why and how do dual users quit vaping? Survey findings from adults who use electronic and combustible cigarettes
Source: Tob Induc Dis. 2021 Feb 16;19:12. doi: 10.18332/tid/132547 (PMC7885258; doi:10.18332/tid/132547)
Supplement: Supplementary file 1 [file TID-19-12-s1.pdf]

Supplemental document to:

Why and how do dual users quit vaping? Survey findings from adults who use electronic and combustible cigarettes

Elias M. Klemperer, Ph.D. & Andrea C. Villanti, Ph.D., MPH

Table S1. Characteristics of excluded participants (n=162)

**Sex**: **Female, % (95% CI)**	32.1 (25.3 to 39.7)
**Mean (SD) age**	35.2 (9.1)
**Marital Status, % (95% CI)**	
Married or living as if married	61.1 (53.3 to 68.4)
Widowed, divorced, separated	4.3 (2.1 to 8.8)
Never married	34.6 (27.6 to 42.2)
**Education, % (95% CI)**	
< High school	1.9 (0.6 to 5.6)
High school graduate or GED	8.6 (5.2 to 14.1)
Some college or Associates degree	20.4 (14.8 to 27.3)
Bachelor's or advanced degree	69.1 (61.6 to 75.8)
**Hispanic ethnicity, % (95% CI)**	13.0 (8.6 to 19.1)
**Race, % (95% CI)**	
American Indian or Alaska Native	1.2 (0.3 to 4.8)
Asian	4.9 (2.5 to 9.6)
Black or African American	8.6 (5.2 to 14.1)
Native Hawaiian or Pacific Islander	1.2 (0.3 to 4.8)
White	84.0 (77.4 to 88.9)
**Employment, % (95% CI)**	
Full-time ≥ 35 hours per week	85.8 (79.5 to 90.4)
Part-time < 35 hours per week	8.6 (5.2 to 14.1)
Don't currently work for pay	5.6 (2.9 to 10.4)
**Past 30 day use**	
Daily EC use	23.5 (17.5 to 30.7)
Daily CC use	8.6 (5.2 to 14.1)

CC=Combustible cigarette; EC=Electronic cigarette; GED=General Education Development; SD=Standard deviation

Table S2. Motivations and methods for quitting e-cigarettes (EC) and combustible cigarettes (CC) among dual users (N=204).

	**ECs**<br>% (95% CI)	**CCs**<br>% (95% CI)
[a]**Motivations to quit**		
Health	74.0 (67.7 to 79.6)	77.5 (71.1 to 82.7)
Money/cost	45.1 (38.4 to 52.0)	44.6 (37.9 to 51.5)
Reduce the risk from COVID-19	24.5 (19.1 to 30.9)	26.0 (20.4 to 32.5)
Increased ECs	-	12.7 (8.8 to 18.1)
Increased other tobacco	20.1 (15.1 to 26.2)	4.9 (2.6 to 26.2)
Freedom from addiction	15.2 (10.9 to 20.8)	17.2 (12.6 to 23.0)
For another person	17.2 (12.6 to 23.0)	19.1 (14.3 to 25.1)
To perform better in school etc	19.1 (14.3 to 25.1)	12.3 (8.4to 17.5)
Negative experience while using	15.7 (11.3 to 21.4)	14.2 (10.0 to 19.7)
Difficulty obtaining	14.2 (10.0 to 19.7)	9.8 (6.4 to 14.7)
Embarrassed by my use	8.8 (5.6 to 13.6)	10.8 (7.2 to 15.9)
Didn't like the taste or smell	11.8 (8.0 to 17.0)	14.2 (10.0 to 19.7)
Didn't like it anymore	7.8 (4.8 to 12.5)	6.9 (4.1 to 11.3)
Other	2.0 (0.7 to 5.1)	1.0 (0.2 to 3.9)
[a]**Methods used to quit**		
Cut down before quitting	68.1 (61.4 to 74.2)	57.4 (50.4 to 64.0)
Cold turkey	24.0 (18.6 to 30.3)	25.0 (19.5 to 31.4)
Got advice from doctor	28.4 (22.6 to 35.0)	33.3 (27.2 to 40.1)
Switched to ECs	-	16.7 (12.1 to 22.5)
Switched to low nicotine pods	23.5 (18.2 to 29.9)	-
Used NRT	24.0 (18.6 to 30.4)	31.9 (25.8 to 38.6)
Read written material	23.0 (17.7 to 29.4)	17.6 (13.0 to 23.5)
Used an app	18.1 (13.4 to 24.1)	5.4 (3.0 to 9.5)
Went to counseling	11.8 (8.0 to 17.0)	14.2 (10.0 to 19.7)
Switched to different tobacco	6.9 (4.1 to 11.2)	3.4 (1.6 to 7.1)
Used buproprion	7.4 (4.5 to 11.9)	14.2 (10.0 to 19.7)
Used varenicline	4.4 (2.3 to 8.3)	7.4 (4.5 to 11.9)
Called a quitline	2.9 (1.3 to 6.4)	3.9 (2.0 to 7.7)
Other	1.0 (0.2 to 3.9)	1.0 (0.2 to 3.9)
[b]**Most effective method used to quit**		
Cut down before quitting	33.3 (27.2 to 40.1)	32.8 (27.6 to 40.8)
Cold turkey	10.8 (7.2 to 15.9)	10.3 (7.0 to 15.8)
Got advice from doctor	13.7 (9.6 to 19.2)	16.7 (12.5 to 23.1)
Switched to ECs	-	5.9 (3.5 to 10.4)
Switched to low nicotine pods	14.2 (10.0 to 19.7)	-
Used NRT	6.9 (4.1 to 11.3)	14.2 (10.3 to 20.3)
Read written material	6.9 (4.1 to 11.3)	4.9 (2.7 o 9.2)
Used an app	4.4 (2.3 to 8.3)	2.5 (1.0 to 6.0)
Went to counseling	4.4 (2.3 to 8.3)	4.9 (2.4 to 8.5)
Switched to different tobacco	2.5 (1.0 to 5.8)	0.5 (0.1 to 3.5)
Used buproprion	0.5 (0.1 to 3.5)	2.5 (1.0 to 5.8)

Used varenicline	0.5 (0.1 to 3.5)	2.0 (0.8 to 5.3)
Called a quitline	1.0 (0.2 to 3.9)	0
Other	1.0 (0.2 to 3.9)	0.5 (0.1 to 3.5)

[a]Respondents were instructed to *"select all that apply"* and thus could select multiple responses.
[b]Respondents were instructed to identify the single method that was *"most effective to help you quit."*
Cold turkey=Quit abruptly without help; COVID-19=2019 Novel coronavirus; NRT=Nicotine replacement therapy. All findings are displayed in Figure 1 of the manuscript.

Table S3. Point-biserial correlations (r_{pb}) between motivations for quitting electronic cigarettes (EC) and combustible cigarettes (CC).

		Reasons for quitting ECs												
		Health	Money/ cost	Taste/ Smell	Difficulty obtaining	Negative experience	Embarrassed	Freedom from addiction	COVID-19	School/ work	Another person	Other tobacco	Didn't like	Other
Reasons for quitting CCs: Health	r_{pb}	**0.536**	-0.006	0.088	-0.015	0.104	-0.039	0.065	0.007	-0.125	-0.159	0.066	0.027	-0.008
	p-value	<.001	0.932	0.212	0.826	0.140	0.580	0.355	0.915	0.074	0.023	0.350	0.707	0.906
Money/cost	r_{pb}	0.149	**0.574**	0.162	0.171	0.020	0.138	-0.023	-0.122	-0.010	0.062	0.214	0.142	0.086
	p-value	0.033	<.001	0.021	0.014	0.780	0.049	0.747	0.083	0.888	0.375	0.002	0.043	0.219
Taste/Smell	r_{pb}	0.049	0.308	**0.461**	0.116	0.210	0.269	0.219	0.029	0.123	0.075	0.076	0.299	0.246
	p-value	0.485	<.001	<.001	0.099	0.003	<.001	0.002	0.679	0.079	0.284	0.280	<.001	<.001
Difficulty obtaining	r_{pb}	-0.143	0.066	0.238	**0.432**	0.220	0.362	0.228	0.080	0.301	0.243	0.205	0.272	0.310
	p-value	0.041	0.351	<.001	<.001	0.002	<.001	0.001	0.253	<.001	<.001	0.003	<.001	<.001
Negative experience	r_{pb}	-0.079	0.139	0.200	0.437	**0.442**	0.220	0.141	0.062	0.302	0.262	0.181	0.247	0.246
	p-value	0.262	0.048	0.004	<.001	<.001	0.002	0.045	0.380	<.001	<.001	0.010	<.001	<.001
Embarrassed	r_{pb}	0.134	0.098	0.314	0.221	0.198	**0.393**	0.249	0.022	0.233	0.177	0.141	0.428	0.293
	p-value	0.056	0.164	<.001	0.002	0.005	<.001	<.001	0.751	<.001	0.011	0.044	<.001	<.001
Freedom from addiction	r_{pb}	0.121	0.032	0.116	0.113	0.054	0.133	**0.423**	0.013	0.010	0.138	0.096	0.254	0.123
	p-value	0.084	0.652	0.098	0.109	0.443	0.057	<.001	0.856	0.885	0.049	0.171	<.001	0.079
COVID-19	r_{pb}	-0.057	0.047	0.096	0.239	0.113	0.170	0.154	**0.650**	0.281	0.175	0.038	0.201	0.158
	p-value	0.419	0.503	0.172	<.001	0.107	0.015	0.028	<.001	<.001	0.012	0.593	0.004	0.024
School/work	r_{pb}	0.085	0.082	0.235	0.319	0.168	0.200	0.092	0.204	**0.351**	0.266	0.186	0.225	0.271
	p-value	0.226	0.244	<.001	<.001	0.017	0.004	0.192	0.003	<.001	<.001	0.008	0.001	<.001
Another person	r_{pb}	0.004	0.085	0.171	0.195	0.065	0.288	0.072	0.042	0.239	**0.605**	0.129	0.229	0.201
	p-value	0.957	0.224	0.015	0.005	0.359	<.001	0.306	0.553	<.001	<.001	0.065	<.001	0.004
Other tobacco	r_{pb}	0.135	0.068	0.340	0.298	0.277	0.410	0.283	0.135	0.236	0.318	**0.283**	0.356	0.459
	p-value	0.055	0.334	<.001	<.001	<.001	<.001	<.001	0.055	<.001	<.001	<.001	<.001	<.001
Didn't like	r_{pb}	0.117	0.144	0.262	0.223	0.256	0.257	0.371	0.071	0.164	0.134	0.251	**0.426**	0.381
	p-value	0.097	0.040	<.001	0.001	<.001	<.001	<.001	0.315	0.019	0.057	<.001	<.001	<.001
Other	r_{pb}	0.059	0.010	0.118	0.102	0.094	0.144	0.096	0.059	0.078	0.087	0.074	0.156	**0.345**
	p-value	0.402	0.889	0.093	0.147	0.182	0.039	0.170	0.402	0.267	0.218	0.291	0.026	<.001

Table S4. Point-biserial correlations (r_{pb}) between methods used to quit electronic cigarettes (EC) and combustible cigarettes (CC).

			Strategies used to quit ECs											
			Reduced	Dr.'s advice	NRT	Buproprion	Varenicline	Quitline	Mobile app	Written material	Counseling	Cold turkey	Other tobacco	Switched to low nicotine pods
Strategies used to quit CCs	Reduced	r_{pb}	**0.580**	0.016	-0.026	0.091	-0.008	0.150	0.123	-0.046	-0.024	0.090	0.038	-0.106
		p-value	<.001	0.819	0.716	0.195	0.912	0.032	0.080	0.513	0.738	0.198	0.589	0.132
	Dr.'s advice	r_{pb}	-0.007	**0.499**	0.114	0.159	0.203	0.123	0.045	0.156	0.226	-0.057	0.096	0.196
		p-value	0.916	<.001	0.106	0.023	0.004	0.079	0.523	0.025	0.001	0.420	0.172	0.005
	NRT	r_{pb}	-0.007	0.175	**0.354**	0.251	0.263	0.255	0.115	-0.024	0.175	0.133	0.147	0.266
		p-value	0.926	0.012	<.001	<.001	<.001	<.001	0.102	0.729	0.012	0.059	0.036	<.001
	Buproprion	r_{pb}	-0.053	0.179	0.100	**0.584**	0.391	0.345	0.173	0.211	0.156	0.100	0.223	0.271
		p-value	0.451	0.010	0.156	<.001	<.001	<.001	0.014	0.002	0.026	0.156	0.001	<.001
	Varenicline	r_{pb}	-0.049	0.197	0.237	0.424	**0.397**	0.396	0.306	0.292	0.247	0.149	0.221	0.242
		p-value	0.485	0.005	0.001	<.001	<.001	<.001	<.001	<.001	<.001	0.033	0.002	<.001
	Quitline	r_{pb}	0.084	0.209	0.182	0.524	0.448	**0.563**	0.298	0.249	0.318	0.300	0.445	0.186
		p-value	0.233	0.003	0.009	<.001	<.001	<.001	<.001	<.001	<.001	<.001	<.001	0.008
	Mobile app	r_{pb}	0.117	0.234	0.221	0.349	0.371	0.472	**0.395**	0.127	0.384	0.171	0.364	0.226
		p-value	0.097	0.001	0.001	<.001	<.001	<.001	<.001	0.070	<.001	0.015	<.001	0.001
	Written material	r_{pb}	0.013	0.164	0.161	0.214	0.214	0.224	0.183	**0.602**	0.110	0.161	0.129	0.107
		p-value	0.854	0.019	0.021	0.002	0.002	0.001	0.009	<.001	0.116	0.021	0.067	0.128
	Counseling	r_{pb}	-0.143	0.241	0.231	0.369	0.254	0.428	0.355	0.244	**0.505**	0.133	0.167	0.271
		p-value	0.041	0.001	0.001	<.001	<.001	<.001	<.001	<.001	<.001	0.059	0.017	<.001
	Cold turkey	r_{pb}	0.079	0.038	0.179	0.141	0.152	0.235	0.228	0.087	0.070	**0.550**	0.112	-0.053
		p-value	0.262	0.593	0.010	0.044	0.030	0.001	0.001	0.214	0.318	<.001	0.111	0.448
	Other tobacco	r_{pb}	0.071	0.180	0.146	0.256	0.222	0.286	0.191	0.089	0.182	0.146	**0.268**	0.213
		p-value	0.312	0.010	0.037	<.001	0.001	<.001	0.006	0.207	0.009	0.037	<.001	0.002
	Switched to ECs	r_{pb}	0.080	-0.019	0.149	0.227	0.352	0.234	0.131	0.161	0.204	0.303	0.243	**0.248**
		p-value	0.255	0.783	0.034	0.001	<.001	0.001	0.062	0.021	0.003	<.001	<.001	<.001

© 2021 Klemperer E.M. and Villanti A.C.
